# Opsonic and Antibody Responses to Pneumococcal Polysaccharide in Rheumatoid Arthritis Patients Receiving Golimumab Plus Methotrexate

**DOI:** 10.1097/MD.0000000000002184

**Published:** 2015-12-31

**Authors:** Kiyoshi Migita, Yukihiro Akeda, Manabu Akazawa, Shigeto Tohma, Fuminori Hirano, Haruko Ideguchi, Ryutaro Matsumura, Eiichi Suematsu, Tomoya Miyamura, Shunsuke Mori, Takahiro Fukui, Yasumori Izumi, Nozomi Iwanaga, Yuka Jiuchi, Hideko Kozuru, Hiroshi Tsutani, Kouichirou Saisyo, Takao Yamanaka, Shiro Ohshima, Naoya Mori, Akinori Matsumori, Kiyoki Kitagawa, Koichiro Takahi, Tetsuo Ozawa, Norikazu Hamada, Kyoichi Nakajima, Hideaki Nagai, Norio Tamura, Yasuo Suenaga, Masaharu Kawabata, Toshihiro Matsui, Hiroshi Furukawa, Kenji Kawakami, Kazunori Oishi

**Affiliations:** From the Japanese National Hospital Organization (NHO), Multi-center Clinical Studies for Evidence-based Medicine Study Group; Japanese Study of Randomized Controlled Study for Patients with RA Using 23-Valent Pneumococcal Polysaccharide Vaccine (RA-PPV23), Meguro, Tokyo (KM, ST, FH, HI, RM, ES, TM, SM, TF, YI, NI, YJ, HK, HT, KS, TY, SO, NM, AM, KK, KT, TO, NH, KN, HN, NT, YS, MK, TM, HF, KK); Research Institute for Microbial Diseases, Osaka University, Suita, Osaka (YA); Department of Public Health and Epidemiology, Meiji Pharmaceutical University, Kiyose, Tokyo (MA); and Infectious Diseases Surveillance Center, National Institute of Infectious Diseases, Shinjuku, Tokyo, Japan (KO).

## Abstract

Vaccination against *Streptococcus pneumoniae* is recommended for rheumatoid arthritis (RA) patients receiving immunosuppressive treatments. The objective of this study was to evaluate the humoral response to 23-valent pneumococcal polysaccharide vaccination (PPSV23) in RA patients receiving methotrexate (MTX) alone or in combination with a tumor necrosis factor inhibitor, golimumab (GOM).

PPSV23 was given to 114 RA patients, who were classified into three groups: RA control (n = 35), MTX alone (n = 55), and GOM + MTX (n = 24). Before and 4 to 6 weeks after vaccination, concentrations of antibodies against pneumococcal serotypes 6B and 23F were measured using an enzyme-linked immunosorbent assay and antibody functionality was determined using a multiplexed opsonophagocytic killing assay, reported as the opsonization index (OI).

The IgG concentrations and OIs were both significantly increased in all treatment groups in response to PPSV23 vaccination. In the GOM + MTX group, the IgG responses were lower than those in the MTX alone or control groups, whereas the OI responses were similar to those in the other 2 groups. Furthermore, discrepancies between the IgG and OI responses were found in GOM + MTX group. No severe adverse effect was observed in any treatment groups.

OI responses indicate that antibody functionality rather than antibody quantity is important. The similarity of these measurements between all 3 groups suggests that RA patients receiving MTX + GOM still benefit from receiving the PPSV23 vaccination, even though they produce less IgG in response to it. These results can help clinicians to better schedule and evaluate pneumococcal vaccination for RA patients.

## INTRODUCTION

Infections cause significant morbidity and mortality in patients with rheumatoid arthritis (RA).^1^ The incidence of infections in RA patients was higher compared to healthy subjects.^2^ The introduction of antitumor necrosis factor (TNF) treatments seems to be related with a altered pattern of infections in RA.^3^ Vaccines are useful for the prophylaxis for various infections.^4^ Pneumococcal capsular polysaccharide vaccine (PPSV23) contains 23 purified capsular polysaccharide antigens of *Streptococcus pneumonia*, which cause invasive pneumococcal infections.^5^ The immunogenicity, efficacy, and safety of PPSV23 should to be addressed in RA patients receiving anti-TNF treatments, since these patients have some immune defects.

A recent meta-analysis suggested that anti-TNF treatment alone has no significant negative impact on the response to PPSV23, while patients receiving a TNF inhibitor plus methotrexate (MTX) seem to have impaired immune responses to PPSV23.^6^ Infections with *S. pneumoniae* are a major cause of mortality and morbidity throughout the world.^7^ Guidelines indicate that vaccination with PPSV23 should be considered in RA patients treated with biologics.^8^ However, the use of immunosuppressive therapies can reduce the response to vaccination.

Golimumab (GOM), a humanized monoclonal antibody against TNF-α is effective and generally well-tolerated when administered in combination with MTX to patients with moderate to severe RA.^9^ The aim of the present study was to establish the influence of GOM in combination with MTX on the antibody response to vaccination with PPSV23. Protective immunity against *S. pneumoniae* is mainly mediated by opsonin-dependent phagocytosis, therefore, the opsonophagocytic activity of antibodies to pneumococcal polysaccharides may reflect their functional activity and may represent more valuable marker for in-vivo protection than the antibody concentration.^10^ Therefore, we measured the antibody opsonophagocytic activity against pneumococcal serotypes 6B and 23F in addition to the IgG concentrations.

## METHODS

### Study Design and Patient Population

We performed a randomized, double-blind, controlled trial. Patients with clinically diagnosed RA were recruited at Japanese National Hospital Organization (NHO) hospitals across Japan (n = 32) from September 2010 to December 2012.^11^ Eligible patients were also found to be at risk for developing respiratory infections. RA patients were divided into the following groups: patients with rheumatoid lung disease, patients with RA treated with biological agents, and patients treated with immunosuppressive agents. Patients who previously received PPSV23 were excluded in this study. This study complied with the principles of the Declaration of Helsinki and was approved by the appropriate institutional review boards at each participating center. All patients provided written informed consent. This study was approved by the ethical committees of NHO central IRB (No. 0512014, 2012) and was registered with UMIN-CTR (UMIN000009566).

## INTERVENTION

Patients were randomly assigned to receive either 0.5 mL (25 μg) of PPSV23 (Pneumovax NP, Merck Sharp & Dohme Corp., Tokyo, Japan) or 0.5 mL of a placebo (sodium chloride) subcutaneously in the upper arm. The vaccines were prepared, and both the administrator and patient were blinded to the type of vaccine given. Vaccine and placebo were presented in identical single dose syringes and needle combinations that were labeled with sequential study numbers only. A statistician who was not on the study team carried out the randomization using a random number table and numbered the containers accordingly.

### Enzyme-Linked Immunosorbent Assays for Serotype-Specific IgG

Blood samples were drawn at vaccination and 4 to 6 weeks thereafter, and stocked at −30°C. Enzyme-linked immunosorbent assays (ELISAs) for serotype-specific IgG were performed to measure the concentration of each type of antibody as previously described.^12^ Furthermore, to measure IgG specific for the 6B and 23F serotypes, we specifically performed our ELISAs according to the World Health Organization (WHO) standard procedure that uses the international reference serum, 89SF-3 (kindly supplied by Dr. Carl E. Frasch). To improve the specificity of the assay, a pneumococcal cell wall polysaccharide (C-PS) and pneumococcal 22F polysaccharide pre-absorption step was performed on the samples. The reference serum was pre-absorbed with C-PS only.^13,14^ Detailed protocols are available at www.vaccine.uab.edu /ELISAProtocol (89SF).pdf.

### Multiplexed Opsonophagocytic Assays

To measure antibody functionality against pneumococcus, we performed multiplexed opsonophagocytic assays (OPAs) for pneumococcal serotypes 6B and 23F, using differentiated HL-60 cells and antibiotic-resistant target bacteria strains, at the Research Institute for Microbial Disease, Osaka University, as previously described.^15^ The quality control serum included in each assay was prepared from pooled sera of adults immunized with 23-valent pneumococcal polysaccharide (PPV23). Opsonization indices (OIs) were defined as the serum dilution that caused 50% death of target bacteria. Opsotiter 3, an excel-based data processing program, was used to convert colony counts to OIs, according to the WHO protocol available at www.vaccine.uab.edu /UAB-MOPA.pdf.

### Antibody Response

Fold increases of IgG concentrations and OIs relative to prevaccination values were determined. A positive antibody response was assessed as more than 2-fold increase in IgG concentrations and as more than 10-fold increase in OIs as described previously.^12^

## Statistical Analysis

The study population was classified into three groups based on the RA treatment at vaccination. Clinical and demographic characteristics of each group were expressed as mean ± standard deviation or as a percentage. Changes in IgG geometric mean concentrations (GMCs) and opsonophagocytic activity (OPA) titers before and after vaccination were compared using a paired-sample *t*-test. To compare categorical variables in response rates between groups, the Pearson Chi-square and Cramer V test were calculated for 2 × 3 tables, respectively. Multivariate logistic regression analysis with adjustment for baseline characteristics was used to assess the relationship between positive antibody response to pneumococcal serotypes and a set of predictor variables including age, sex, current MTX, prednisolone use, GOM use, lymphocyte counts, and serum IgG levels. A backward stepwise selection procedure was used to select significant independent variables. For all tests, probability values (*P* values) less than 0.05 were considered statistically significant. All calculations were performed using Excel Statistical Analysis 2008 (SSRI Co., Ltd., Tokyo, Japan) or PASW Statistics version 20 (SPSS Japan Inc., Tokyo, Japan).

## RESULTS

### Clinical and Demographic Characteristics

**FIGURE 1:**
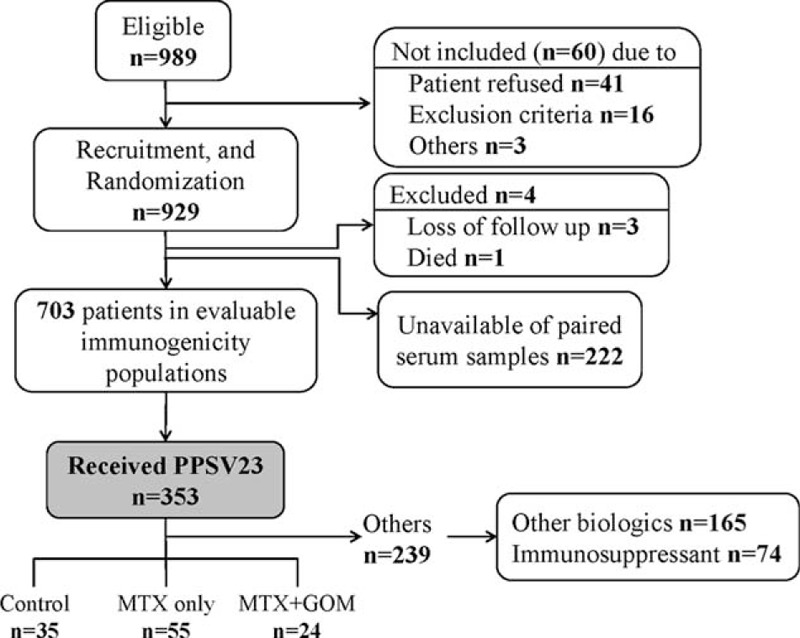
Flow diagram of patient recruitment.

A total of 989 RA patients were assessed for eligibility, and 929 patients were recruited and randomized. Of these, paired serum samples were obtained before and after vaccination from 703 patients, 353 of whom received PPV23. We selected a study population who had received PPSV23 and ongoing anti-RA therapy of disease-modifying antirheumatic drugs (DMARD), MTX, and/or GOM to analyze the impact on immune responses (Fig. 1). The study population was classified into 3 groups: DMARD treatment only (RA control group; n = 35); MTX monotherapy (MTX group, n = 55); and MTX plus GOM treatment (n = 24), and their clinical and demographic characteristics are summarized in Table 1. All patients fulfilled the criteria of safety required for vaccine injection, and no serious side effects were observed after vaccination.

**TABLE 1:**
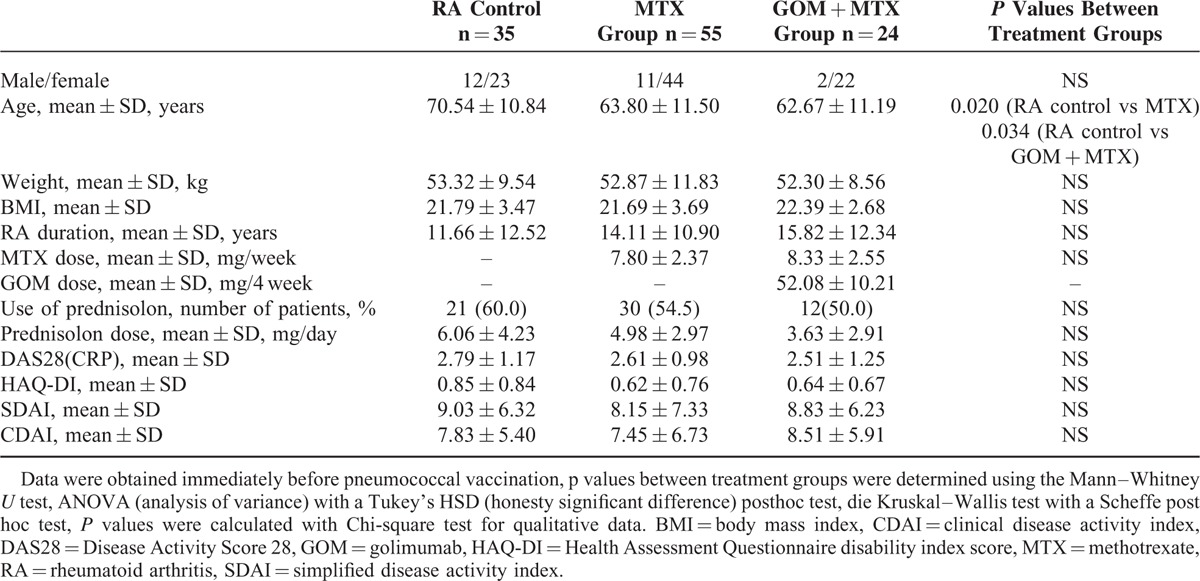
Clinical and Demographic Characteristics of RA Patients Prior to Pneumococcal Vaccination

### Serotype-Specific IgG Concentrations

The ratios between post- and prevaccination antibody concentrations are summarized in Table 2. After PPSV23 vaccination, the GMCs of both serotype 6B- and 23F-specific IgG were significantly increased in all groups. There were large differences in the fold induction of GMC responses among the groups with regard to treatments. For both serotypes, a higher post-GMC was obtained in the control (2.38-fold in 6B and 3.36-fold in 23F) and MTX alone (1.75-fold in 6B and 2.00-fold in 23F) groups compared with that in the GOM + MTX (1.23-fold in 6B and 1.41-fold in 23F) group.

**TABLE 2:**
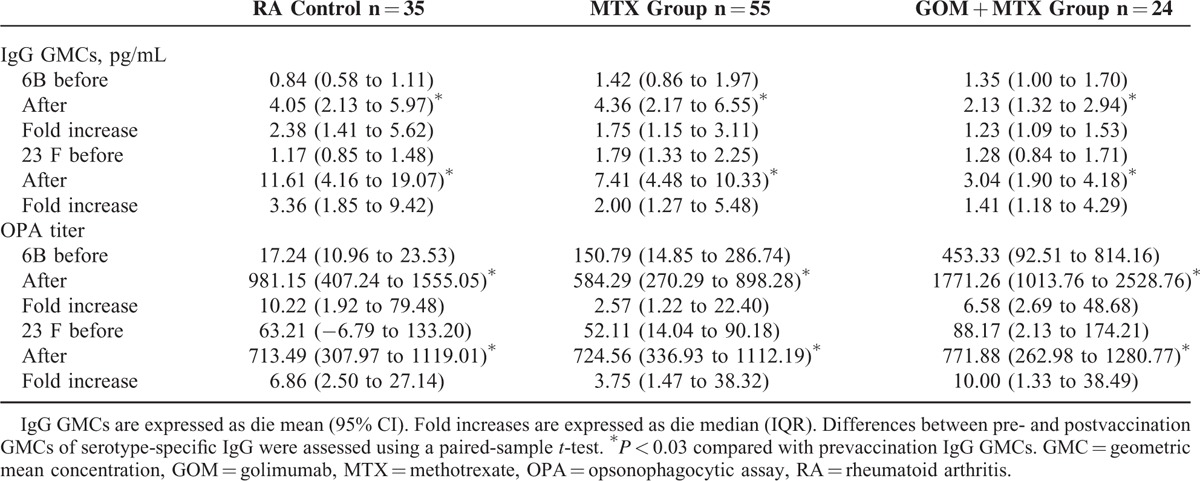
Concentrations of Pneumococcal Polysaccharide Antigen Serotype-Specific IgG Antibodies and Opsonization Indices in the RA Treatment Groups Before and After 23-Valent Pneumococcal Polysaccharide Vaccination

### Opsonophagocytic killing Assays

The postvaccination OIs were significantly increased in all treatment groups. The ratios between pre-and postvaccination are shown in Table 2. In contrast to the GMC results, there was no difference in the fold induction of OIs among the GOM + MTX, and control or MTX groups. Using this assay, the GOM + MTX group showed an antibody response rate that was equivalent to that in the control or MTX groups.

### Antibody Response Rates

**FIGURE 2:**
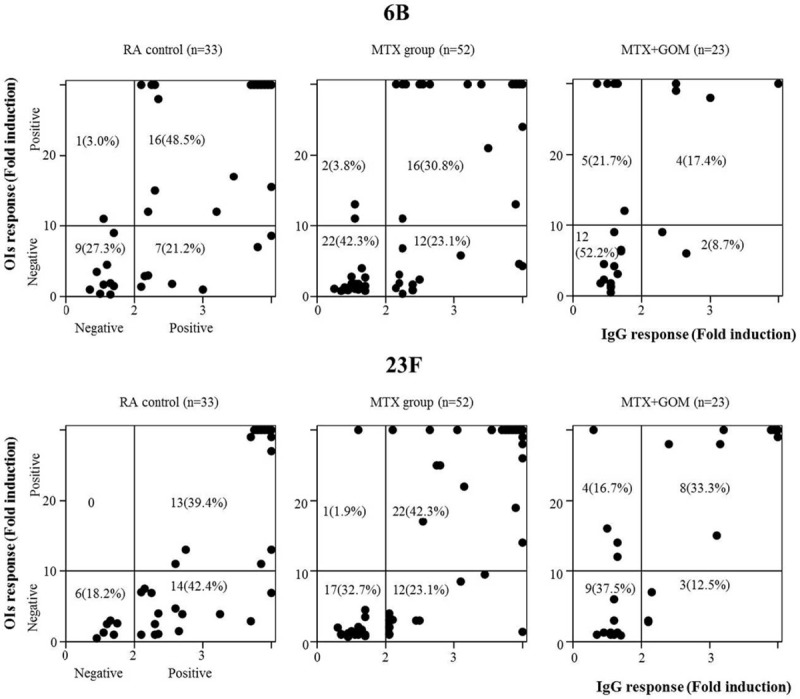
Comparison of postvaccination GMC responses in patients receiving MTX and MTX plus GOM. Percentages of patients with an increase in 6B and 23F serotype-specific IgG concentration greater than 2-fold were shown. There were significant differences in the response rates among control, MTX alone and MTX plus GOM (Cramer's V test *P* < 0.012). There were significant differences in the response rates between MTX plus GOM and control or MTX alone. Data were compared using the Pearson Chi-square test. GMC = geometric mean concentration, GOM = golimumab, MTX = methotrexate.

**FIGURE 3:**
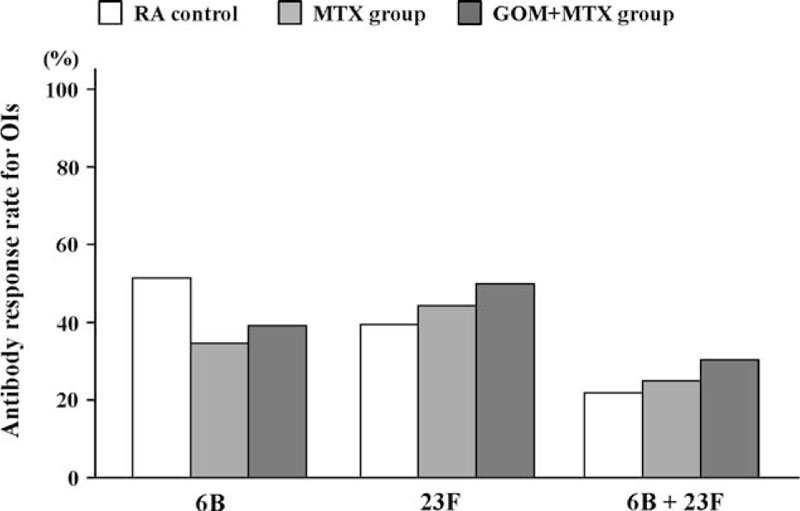
Comparison of postvaccination OIs responses in patients receiving MTX and MTX plus GOM. Percentage of patients with an increase in OIs for serotypes 6B and 23F greater than 10-fold were shown. There were no significant differences in the response rates among control, MTX alone and MTX plus GOM (Cramer V test). There was no significant difference in the response rates between MTX plus GOM and control or MTX alone. Data were compared using the Pearson Chi-square test. GOM = golimumab, MTX = methotrexate, OI = opsonization index.

The GMC response rates, given as the percentage of patients with a positive antibody response, for patients in the GOM + MTX group were significantly decreased compared with those of the control or MTX groups for both serotype 6B and 23F (Fig. 2). For OPIs specific for serotype 6B and 23F, the GOM + MTX group showed an equivalent antibody response rate, similarly defined as the percentage of patients with a positive antibody response, compared with the control or MTX groups (Fig. 3). Because the OPA is a measurement of antibody function, these results suggest that while the GMC response rate is lower in patients receiving MTX + GOM, the antibodies produced in response to PPSV23 vaccination by this group have a similar functional ability.

### Associations Between IgG and OIs Responses

**FIGURE 4:**
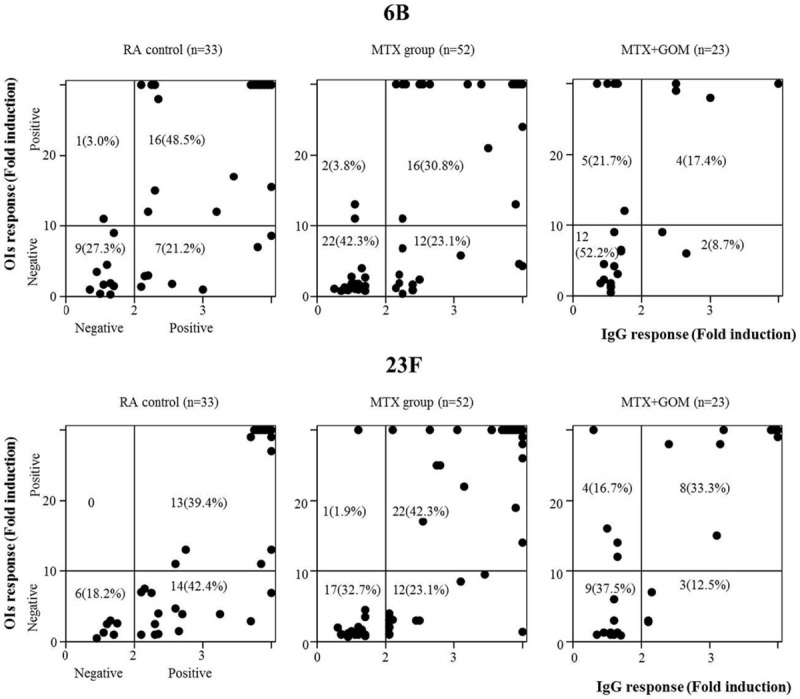
Relationship between IgG and OIs responses after PPSV23 vaccination. The responses 6B (upper panel) and 23F (lower panel) serotype-specific IgG (X-axis) and OIs (Y axis) responses were plotted in the comparison of three groups (control, MTX, MTX + GOM). Positive OIs responses were demonstrated in patients receiving MTX plus GOM with negative IgG responses. GOM = golimumab, MTX = methotrexate, OI = opsonization index.

Finally, we assessed potential associations between IgG and OIs responses for serotype 6B and 23F in each treatment group. In the control and MTX groups, the functional OI response was almost exclusively observed in patients with optimum IgG responses. In contrast, in the MTX plus GOM group a positive OI response was also observed in patients who lacked an optimum IgG response (Fig. 4). Therefore, an association between IgG responses and OI responses was not demonstrated in patients receiving MTX plus GOM.

### Predictive Factors for Antibody Responses to PPSV23

In a multivariate logistic regression analysis, GOM use was identified as the predictive factor for reduced IgG responses to both serotypes 6B and 23F (Table 3). However, a negative association of GOM use with OI responses was not confirmed for both concentrations of IgG specific for serotypes 6B and 23F (Table 4).

**TABLE 3:**
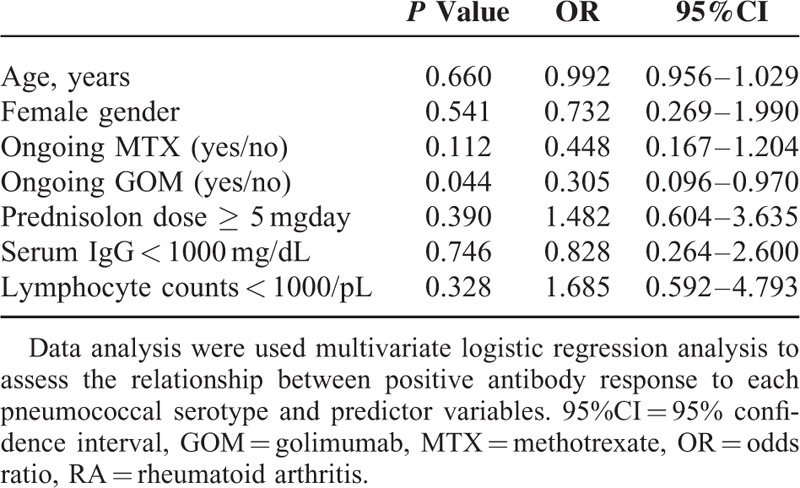
Predictors of Positive IgG response for Both 6B and 23F Among RA Patients

**TABLE 4:**
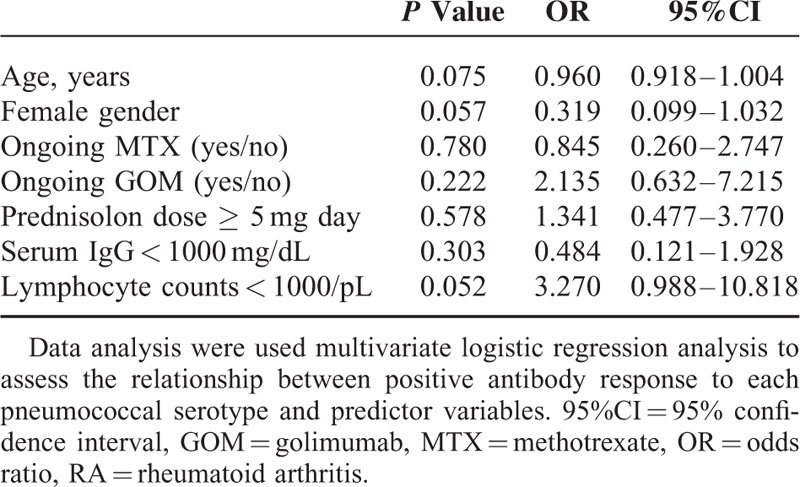
Predictors of Positive OLs Response for Both 6B and 23F Among RA Patients

## Safety

There were no reported adverse events associated with PPSV23 vaccination in the patients from this study.

## DISCUSSION

The aim of this study was to investigate the effects of GOM treatment during MTX therapy on the immune responses to PPSV23 vaccination by determining IgG levels and the levels of functional antibodies (opsonophagocytic avidity). In patients receiving both drugs, the IgG response rates were lower than those in control group patients, who received DMARDs or in patients who received MTX alone. In PPSV23-vaccinated RA patients, GOM + MTX treatment had a suppressive effect on the subsequent GMC responses, but not on the OI responses, compared with other DMARD treatment or treatment with MTX alone. Differences between the IgG and OI responses were found mainly in the GOM + MTX group. We concluded that the IgG response induced by PPSV23 was impaired; however, OI responses after pneumococcal vaccination were preserved in patients receiving combined MTX plus GOM therapy.

Several studies demonstrated that humoral immune responses to polysaccharide pneumococcal vaccines are maintained in RA patients receiving treatment with anti-TNF therapy.^16^ The single use of adalimumab did not affect antibody titers against pneumococcal serotypes;^17^ however, treatment with a combination of MTX and adalimumab decreased response rates compared with MTX treatment alone.^18^ Similarly, although humoral immune responses to pneumococcal vaccination were not impaired by certolizumab pegol (CZP) treatment, vaccine responses were reduced by CZP treatment with concomitant MTX use.^19^ These studies suggested that the immune response to pneumococcal polysaccharide vaccination can be impaired in patients treated with MTX in combination with some TNF antagonists, which are known to have immunosuppressive effects.^6^ Our data also indicated that humoral immune responses to PPSV23 were impaired in patients treated with a humanized monoclonal anti-TNF, GOM, in combination with MTX. However, our study assessing the PPSV23 immunogenicity in patients receiving GOM + MTX demonstrated discrepancies between IgG concentrations and functional anti-pneumococcal OPA in these patients. Previous investigations indicated that tocilizumab or tacrolimus monotherapy did not reduce IgG or opsonic responses to PPSV23 vaccination in patients with RA.^11,12^ MTX was associated with reduced IgG responses to PPSV23.^6^ Therefore, IgG responses to PPSV23 might be impaired in patients treated with GOM + MTX. However, GOM + MTX did not affect OI responses to PPSV23 vaccination.

Components that contribute to OPA are antibody characteristics, such as concentration, isotype, avidity, and serotype-specific IgG concentration.^20^ Recent evidence indicated that older adults have a lower capacity to opsonize pneumococci despite normal IgG levels, because of a lack of anti-pneumococcal IgM antibodies.^21^ The poor correlation between opsonic activity and IgG levels was also shown in patients who are in immunosuppressive states.^22^ RA patients receiving GOM + MTX showed a similar dissociation between opsonic activity and levels of IgG against the polysaccharide capsule of pneumococci. There is growing evidence that anti-TNF treatment may influence the B cell response to antigen challenge.^23^ A recent study demonstrated that anti-TNF treatment impaired T cell-dependent antibody responses and moderately decreased T cell-independent antibody responses after PPV vaccination by affecting B cell activation and maturation.^24^ The mechanisms by which the combined use of GOM + MTX impair the antibody response to PPSV23 vaccination are not known; however, these findings suggest that the immune suppressive effect caused by the combination of GOM + MTX may act on processes that are unique for the T cell-independent polysaccharide vaccine, PPSV23. Functional antibody levels, measured by OPA, provide valuable information for serotype-specific protection in pneumococcal infection.^25^ It is clear that OPA can be performed with the reproducibility and high throughput required for immunogenicity analysis of pneumococcal vaccination.^26^ We propose that determining OPA after PPSV23 vaccination should be required to measure the functionality of this vaccination, especially in patients receiving biologics that may induce a discrepancy between GMC and OPA. Immunogenicity studies comparing PPSV23 and pneumococcal conjugate vaccines 7 (PCV7) revealed that the OPA and geometric antibody titers PCV7 were comparable or higher than those of PPSV23.^27^ Furthermore, a recent cohort study indicated that vaccination with PCV7 tended to reduce the risk of putative serious pneumococcal infections by approximately 45% compared to nonvaccinated patients with inflammatory arthritis.^28^ The Advisory Committee on Immunization Practice (ACIP) recommended the use of both PPV23 and PCV13 for adults aged ≥19 years with immune-compromising conditions, preferably PCV 13 first, followed by PPSV23, or for patients who previously received ≥1 dose of PPSV23 to be administered a dose of PCV13.^29^ The introduction of pneumococcal conjugate vaccines may expand the options available for protecting RA patients against pneumococcal infections. New trials are attempting to clarify the best vaccination strategies to protect against pneumococcal diseases.

The primary limitation of this study was the relatively small number of RA patients in each group, particularly for the GOM + MTX combination treatment. Furthermore, we chose to investigate serotypes 6B and 23F because they are the main causative serotypes of penicillin-resistant pneumococcal pneumonia in Japan.^5^ Although this allowed us to focus solely on these important serotypes, the effects of GOM on other serotypes during the PPSV23 vaccine-induce immune response are still unknown. Lastly, the antibody concentrations necessary for protection against invasive pneumococcal disease in adults have not been clearly defined.^30^ Although we used thresholds indicated by previous studies, namely a 2-fold increase in IgG concentration and a 10-fold increase in the OI, to measure the positive antibody response to PPSV23 in this study, the suitability of these thresholds to predict prevention of pneumococcal infection has not been widely investigated.

In conclusion, we demonstrated an impaired serotype-specific IgG response in patients treated with GOM + MTX after primary vaccination with PPSV23. In contrast, serotype-specific OPI responses were not impaired in these patients compared with those in control patients or patients receiving MTX alone, so there were discrepancies between the GMC and OPI responses after PPSV23 vaccination in patients receiving GOM + MTX. Awareness of these results may be useful to clinicians and enable them to schedule pneumococcal vaccination for RA patients according to their treatments. Furthermore, the functional OPA should be monitored, in addition to serotype-specific IgG concentrations, in patients receiving a TNF blocker plus MTX.

### Ethics Approval

The study was approved by the ethical committees of NHO central IRB (No. 0512014, 2012).

Trial registration: University Hospital Medical Information Network Clinical Trials Registry: UMIN000009566. Registered December 12, 2012.
